# Thinking Outside the Box: A Case of Persistent Symptomatic Hypomagnesemia

**DOI:** 10.7759/cureus.54260

**Published:** 2024-02-15

**Authors:** Rita Relvas, Nuno Ferreira Monteiro, Rita Vale Rodrigues, Diogo Cruz

**Affiliations:** 1 Internal Medicine, Hospital de Cascais, Lisbon, PRT; 2 Gastroenterology, Hospital de Cascais, Lisbon, PRT

**Keywords:** supplementation, malabsorption, iatrogenesis, proton pump inhibitors, hypomagnesemia

## Abstract

Although present in a significant number of people, hypomagnesemia is still an undervalued diagnosis. Therefore, its awareness and comprehensive etiological investigation become imperative. Among its multiple possible causes, drug iatrogenesis plays an important and often overlooked role. Here, we present a case of a 78-year-old female with recurrent bouts of severe hypomagnesemia of unknown origin, which, after an extensive study, was determined to be induced by proton pump inhibitors (PPIs). As such, our goal is to raise awareness of the potential risk of this side effect even in monotherapy, as well as to elucidate its underlying mechanisms, which are still not fully understood. Furthermore, it is intended to foster a systematic therapeutic review in these patients and raise discussion about the potential benefits of systematic magnesium monitoring in patients on long-term PPIs.

## Introduction

Hypomagnesemia affects approximately 7% to 12% of all hospitalized patients and can be as prevalent as 60% to 65% in intensive care units (ICUs) [1,2]. However, its evaluation is not routinely carried out, and given the nonspecificity of its presentation, a strong degree of suspicion for the diagnosis is necessary.

Considering its high prevalence, potential complications, and/or fatal outcomes, accurate diagnosis is imperative so that adequate treatment can be provided. Depending on the cause behind the depletion, it may require a direct intervention on the etiology (e.g., corticoids or immunomodulatory therapy in inflammatory bowel disease [IBD]) or suspension of specific medications, besides the likely need for supplementation (oral or intravenous [IV]) [1].

One possible etiology, still not fully understood, pertains to proton pump inhibitor (PPI)-induced hypomagnesemia. Even though the use of PPI and diuretics in combination has been reported to result in up to 55% increased odds of hypomagnesemia compared with patients on diuretics alone [3], the independent risk of PPI by themselves remains unknown [4].

The authors present a case of persistent, severely symptomatic hypomagnesemia, the root cause of which had remained unclear for several years. The true etiology proved challenging to ascertain due to multiple confounding factors.

Considering the high frequency of PPI treatment worldwide, we aim to raise awareness of a possible and serious adverse effect, potentially warranting more in-depth studies. We also appeal to the rational use of this drug class.

## Case presentation

A 78-year-old female with a past medical history of primary hypertension, dyslipidemia, and gastroesophageal reflux disease secondary to a large hiatal hernia presented. The patient was medicated with amlodipine/valsartan, pitavastatin, and esomeprazole.

She had a history of recurrent hospitalizations over the previous five years due to symptomatic severe hypomagnesemia, presenting with neurological symptoms, including reduced level of consciousness and seizures. Each of these episodes was preceded by a bout of acute viral enteritis with associated diarrhea, leading to magnesium depletion through gastrointestinal (GI) losses. After IV supplementation, the patient would be discharged with serum magnesium levels within the normal range.

The patient presented once more to the emergency department (ED) with tonic-clonic seizures following five days of acute gastroenteritis before hospital admission, with significant diarrhea. The initial workup showed severe magnesium depletion (0.2 mg/dL; normal range 1.6-2.6 mg/dL), with no other relevant changes.

Considering the recurrent nature of symptomatic hypomagnesemia, the patient was admitted to the infirmary for further study after magnesium levels were corrected with IV infusion. Alcohol consumption, bowel surgeries, irregular dietary habits (including ketogenic diets and intermittent fasting), eating disorders (anorexia nervosa or bulimia), dysglycemia, and known family history of ion disturbances were excluded.

Given the history of frequent episodes of acute diarrhea, GI causes were the first to be explored. Upon admission to the ED, the initial physical examination brought attention to light facial flushing, which subsided shortly thereafter. Nevertheless, this, coupled with recurrent bouts of diarrhea, raised suspicion regarding a possible neuroendocrine tumor. To corroborate this hypothesis, chromogranin A, as well as norepinephrine and adrenaline in a 24-hour urine sample, were slightly elevated. However, measurements of gastrin, serotonin, and vasoactive intestinal peptide (VIP) were all within the normal range. A second, later measurement of chromogranin A also fell within the normal range. Additionally, thoracoabdominopelvic computerized tomography (CT) and positron emission tomography-CT (PET-CT) with FDG-F18 revealed no abnormal findings. For further clarification, PET-CT with DOTATOC-Ga68 was also performed, which excluded findings compatible with neuroendocrine tumors, ruling out this diagnosis.

Searching for a possible malabsorptive nature, serologies for celiac disease (anti-endomysial IgA, antigliadin IgA and IgG, transglutaminase IgA and IgG, and total IgA) and autoimmune gastritis (anti-parietal cell and anti-intrinsic factor) were requested. However, these etiologies were excluded after obtaining normal results. Possible GI tract infections were also considered, with negative cytomegalovirus (CMV) and Epstein-Barr (EBV) IgM, as well as negative stool cultures for *Shigella *spp., *Campylobacter jejuni*, and *Salmonella* spp. Normal fecal fat and pH measurements, along with normal ADEK vitamin levels, also excluded pancreatic causes. Nevertheless, an elevated fecal calprotectin (134 µg/g; normal level <50 µg/g) raised suspicion for an inflammatory bowel disease (IBD), necessitating endoscopic studies. However, the timing of these studies was hampered by availability limitations.

Urinary measurements revealed proper electrolyte levels (magnesium, calcium, sodium, phosphorus, and chloride), making a renal cause unlikely. Furthermore, considering a possible endocrine origin, we analyzed phospho-calcium metabolism (parathyroid hormone [PTH], phosphorus, calcium, and albumin), ACTH, and cortisol levels, which were also normal.

At this point, after IV magnesium administration and continued oral supplementation, magnesium levels remained within normal range for an extended period. At this point, an attempt at withdrawing PPI therapy was met with a worsening of reflux symptoms, and as such, esomeprazole had to be restarted. Given continued clinical stability, absence of new seizures or diarrhea, no GI tract symptoms for several days, and sustained levels of serum magnesium, the patient was discharged home with a short-term appointment scheduled. She was instructed to add magnesium pidolate to her habitual therapeutic list. At the time of discharge, endoscopic studies were yet to be performed.

**Figure 1:**
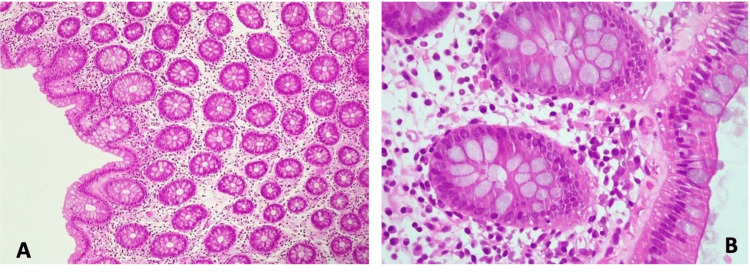
Colonoscopic study: histological images of colon samples using hematoxylin and eosin staining. Colon sample from colonoscopy, with magnification (A) x20 and (B) x40, showing slight collagenous thickening of the basement membrane of the epithelium, not significant, without lymphoid infiltrate - histological findings not meeting microscopic colitis (collagenous or lymphocytic) criteria.

The patient was re-evaluated two weeks later in an outpatient setting, completely asymptomatic, with a serum magnesium level still within the normal range but lower than upon hospital discharge (1.6 mg/dL). The pending esophagogastroduodenoscopy (EGD) revealed only the already-known hiatal hernia, with colonoscopy showing light nonspecific colitis, not meeting microscopic colitis criteria (Figures 1A-1B)]. At this stage, an increase in oral supplementation was made.

Three months later, the patient stood asymptomatic, with no new GI disturbances, but with a drop of serum magnesium to 1.2 mg/dL. Due to the downward kinetics of serum magnesium, the patient received another IV supplementation, maintaining oral supplementation afterward.

At this point, with worsening hypomagnesemia for the first time unrelated to GI losses, the diagnosis of pharmacological iatrogenesis gained strength. Despite a previously unsuccessful attempt, PPI was stopped a second time. Concomitantly, as there have been rare cases of celiac enteropathy-like and lymphocytic colitis associated with angiotensin II receptor blockers (ARBs), valsartan was replaced with lisinopril. This approach, however, led to a resurgence of severe heartburn, yet with a concomitant sustained increase in serum magnesium of around 1.6 mg/dL. For this reason, the H2-receptor antagonist famotidine was introduced as an alternative to esomeprazole.

Concerning the nonspecific colitis, and after discussion with a gastroenterology specialist, the additional investigation included repetition of fecal calprotectin measurement, which was increased (452 µg/g). A three-month trial of oral budesonide was suggested, given that it was not possible to fully exclude an inflammatory and/or malabsorptive etiology. During this period, which coincided with that of esomeprazole and valsartan withdrawal, the patient maintained stable magnesium levels (1.7-1.9 mg/dL). However, shortly thereafter, she reported persistently worsening heartburn even under famotidine, sucralfate, and metoclopramide.

These symptoms led to the decision to start esomeprazole and refer the patient to a General Surgery appointment, aiming for hiatal hernia correction. However, despite an immediate improvement in the GI symptoms, there was a significant fall in magnesium levels, down to 1 mg/dL.

A new trial period of oral budesonide yielded no benefit, and consequently, was suspended. Another IV supplementation was necessary. As the likelihood of an iatrogenic cause was undoubtedly becoming higher, the PPI was again replaced, this time by an antacid (a combination of aluminum hydroxide, magnesium hydroxide, and simethicone). During this period, under suspension of PPI, magnesium levels normalized (1.6-1.7 mg/dL).

With all other possible diagnoses excluded, and the undeniable relation between PPI therapy and worsening magnesium levels, PPI-induced hypomagnesemia became the most likely diagnosis. Chronic use in this patient led to persistently reduced basal magnesium levels, which worsened considerably with each bout of acute enteritis with diarrhea, precipitating severe depletions and its associated symptoms. Moreover, the extensive study for intestinal diseases excluded other causes of chronic diarrhea. In this particular case, the increased fecal calprotectin was an undeniably confounding factor that complicated the diagnosis, as it may - or may not - be related to GI disease (particularly IBD). Nevertheless, its value is not only proven to be increased in other settings but also carries a risk of false positives.

Regardless of the diagnosis made, given the recurrence of disabling heartburn and gastroesophageal reflux, refractory to any other therapeutic option, and aiming to balance risk/benefit, there was a need to reintroduce esomeprazole at the absolute minimum effective dose (once daily, every other day). Since then, the patient continues to be managed in an ambulatory setting with adequate symptom control. Monthly magnesium monitoring is maintained along with oral supplementation, with IV supplementation only sporadically required. The patient has also sought a General Surgery consultation and is currently under evaluation for hiatal hernia correction surgery, to facilitate definitive withdrawal of PPI treatment.

## Discussion

As the second most common intracellular cation, magnesium is responsible not only for several cellular and enzymatic functions but also for the maintenance of vascular tone, muscle contraction and relaxation, bone formation, and nerve and cardiac conduction [2,5]. Additionally, it has a direct impact on the balance of other electrolytes, namely sodium, potassium, and calcium [5]. It is estimated that hypomagnesemia is present in 2.5%-15% of the population, particularly hospitalized patients, ICU patients, alcoholics, and diabetics [1,6,7].

Hence, its depletion has a multiorgan affection, with cardiovascular and neuromuscular manifestations being the most frequent [2,5,6]. Initially, hypomagnesemia may lead to nausea, vomiting, anorexia, fatigue, and muscle weakness, which can quickly progress to neuromuscular hyperexcitability symptoms, manifested by tremor, tetany, seizures (as in our patient), choreoathetosis, nystagmus, apathy, agitation, depression, delirium, psychosis, and even coma [2,5]. Simultaneously, early findings of hypomagnesemia on electrocardiography include widening of the PR interval and QRS complex, as well as spiculated or, if severely deficient, flattened T-waves. These changes pose a risk of extrasystole, atrial fibrillation, ventricular tachycardia, bradycardia, hypotension, and cardiac ischemia [8]. Also, hypokalemia, hypocalcemia, hyperparathyroidism, and parathyroid hormone (PTH) resistance can be common findings, and there is a known increased risk of nephrolithiasis, hypertension, insulin resistance, and metabolic syndrome [9,10].

Magnesium homeostasis is mainly controlled by three organs: the bowel, responsible for its absorption from the diet; bones, the main storage organ (50%-60%); and kidneys, responsible for its excretion [2,5]. Thus, hypomagnesemia results from an anomaly in, at least, one of these organs, whether due to pathology or drug iatrogenesis.

There are several causes of hypomagnesemia, listed in Table 1, and commonly divided into gastrointestinal and renal losses, with other causes being less common [1,2,5]. In the first group, the most common etiologies are diarrhea, malabsorptive syndromes, IBD, acute pancreatitis, post-bariatric surgery status, genetic causes, and drug iatrogenesis, where PPIs stand out as possible agents. As for renal losses, these most often result from drug iatrogenesis, namely diuretics, antibiotics, digoxin, calcineurin inhibitors, or monoclonal antibodies against the epidermal growth factor (EGF) receptor [1,2,6,9]. Other etiologies include hypercalcemia, uncontrolled diabetes mellitus, hereditary tubular dysfunction (such as Gitelman or Bartter syndrome or familial hypomagnesemia with hypercalciuria and nephrocalcinosis), or acquired causes (following acute tubular necrosis or kidney transplantation) [1,2,9]. Less commonly, the causes include anorexia nervosa, starvation, total parenteral nutrition, neuroendocrine tumors, hungry bone syndrome, chronic alcoholism/alcohol withdrawal syndrome, or ketogenic diets [9].

**Table 1:** Hypomagnesemia etiologies. ARB, angiotensin II receptor blocker; EGF, epidermal growth factor; PPI, proton pump inhibitor

Gastrointestinal causes
Diarrhea
Malabsorption syndrome/small bowel bypass surgery
Inflammatory bowel disease
Acute pancreatitis
Medications
- PPI
- ARB
- Laxatives
Genetical disorders
- Intestinal hypomagnesemia with secondary hypocalcemia
Renal causes
Medications
- Diuretics (loop and thiazide)
- Antibiotics (aminoglycoside, amphotericin, pentamidine)
- Calcineurin inhibitors
- Cisplatin
- Digoxin
- Anti-EGF (cetuximab, panitumumab, matuzumab)
Volume expansion
Uncontrolled diabetes mellitus
Hypercalcemia
Acquired tubular dysfunction
- Post-obstructive diuresis
- Post-kidney transplantation
- Post-acute tubular necrosis
Genetic disorders
- Bartter/Gitelman syndrome
- Familial hypomagnesemia with hypercalciuria and nephrocalcinosis
- Autosomal dominant/recessive isolated hypomagnesemia
- Renal malformations
Other causes
Anorexia nervosa/starvation
Chronic alcoholism/alcohol withdrawal syndrome
Total enteral nutrition
Metabolic acidosis correction
Hungry bone syndrome
Ketogenic diets

In the reported case, we conducted an extensive study with a sequential approach, focusing on the more likely hypotheses within the individual context of our patient. Only after excluding other potential causes and considering the impact of PPI therapy on magnesium serum levels did the most likely etiology emerge as iatrogenesis due to its chronic use.

First described in 2006 [11] and subsequently explored, it is known that the risk of PPI-induced hypomagnesemia is particularly high when combined with diuretics. Multiple studies have described this association, with some even attributing PPI-induced hypomagnesemia exclusively to this dual therapy [3,12]. It is reported that daily PPI therapy in conjunction with diuretics is associated with a 43% increased relative risk of hospitalization due to hypomagnesemia. This risk is not observed with more sporadic use of PPIs or with histamine H2 receptor antagonists [12]. However, the evidence for PPI-induced hypomagnesemia alone is still mainly limited to case reports and small case series, which highlight the potential existence of an individual risk, corroborated by this case [13-15]. For instance, a meta-analysis attributes an odds ratio of 1.78 (with a 95% confidence interval of 1.08-2.92) to PPI-induced hypomagnesemia alone, suggesting this class of drugs may indeed constitute an independent risk factor, albeit with some heterogeneity between studies that still prevents definitive conclusions [4].

Despite this assumption, the underlying mechanism is not yet fully established and appears to be related to the deficient absorption of magnesium by the intestinal epithelium [4,12-14]. Physiologically, intestinal magnesium absorption is carried out by two transport systems [2,5]. On the one hand, a transcellular active transport mechanism, via transient potential receptor channels of the melastatin type six and seven (TRPM6 and TRPM7), is particularly important at low luminal magnesium concentrations, increasing fractional magnesium absorption to maximize adaptation to low intake. On the other hand, there is a paracellular passive transport that operates through a simple diffusion mechanism, modulated by tight junctions composed of proteins claudin-16 and 19, which absorb a constant fraction of ingested magnesium (approximately 7%), allowing a linear absorption with luminal magnesium levels [14]. PPIs seem to interfere with active absorption by inhibiting TRPM6 and TRPM7, which is corroborated by the better response to IV than oral supplementation in these patients, as was the case in our patient [3,13-15]. The exact mechanism behind this inhibition and underexpression is still not clear, given that the regulation of these transporters is not yet understood. So far, it is known that TRPM6/7 differs from other ion channels as they are composed of linked channel and protein kinase domains. Moreover, it seems possible that the intestinal pH change induced by PPIs can affect either the enzyme or channel functions [14]. Despite this fact, it is not clear if all PPI users are potentially susceptible or if this can be an idiosyncratic reaction, seen in certain subjects with TRPM6/7 mutations, with a hereditary predisposition being advocated [13,14]. Although the association of other drugs with hypomagnesemia is mainly mediated through increased renal magnesium loss, there is no evidence that PPIs interfere with the renal excretion of magnesium, with it being appropriately low to minimize electrolyte loss, which was also documented in our patient [15]. Finally, this appears to be a drug class effect shown to exist in situations of continuous, chronic use [13,14]. Yet, intermittent PPI use has still not been proven to have such an effect, making its use every other day a potentially viable option in case of PPI dependence for symptom control, as was the case with our patient [12,14].

Regardless of the previously described severe consequences of its depletion, magnesium measurement is still not performed on a routine basis and the recognition of its deficiency requires strong clinical suspicion. Therefore, it is crucial not only to recognize its symptoms but also to maintain close surveillance in situations where, due to the recognized causality, the risk may increase, including the use of PPIs. In fact, in February 2011, the Federal Food and Drug Administration (FDA) published a safety alert regarding patients expected to receive long-term PPI therapy and/or PPI therapy in association with other potentially hypomagnesemia-inducing drugs [16]. This warning suggests not only measuring magnesium levels before starting PPIs and serially during their use but also reserves its use for those who need it (gastroesophageal reflux disease, peptic ulcer disease, or concomitant* Helicobacter pylori *treatment), with the minimum effective dose and period being required and periodically accessed. Even so, the under-recognition of hypomagnesemia remains a reality that needs to be actively tackled.

## Conclusions

Hypomagnesemia affects a significant percentage of people around the world, especially in hospital settings. Although it is associated with serious adverse effects, their nonspecificity makes recognition difficult, leading to considerable misdiagnoses. The authors present a case that illustrates the need to analyze all the individual risk factors, aiming for a structured approach that explores the most likely etiologies in the context of each patient, including a cautious revision of the current medications, their possible side effects, and interactions. Furthermore, the independent risk of PPI-induced hypomagnesemia is emphasized in the presented case, suggesting that, given its worldwide and very common use, further large-scale prospective cohort studies might be useful to corroborate this causality more accurately and elucidate its underlying mechanisms.
